# Psychometric Properties of the Nine-Item Problematic Internet Use Questionnaire (PIUQ-9) in a Lithuanian Sample of Students

**DOI:** 10.3389/fpsyt.2020.565769

**Published:** 2020-11-12

**Authors:** Julius Burkauskas, Orsolya Király, Zsolt Demetrovics, Aurelija Podlipskyte, Vesta Steibliene

**Affiliations:** ^1^Laboratory of Behavioral Medicine, Neuroscience Institute, Lithuanian University of Health Sciences, Palanga, Lithuania; ^2^Institute of Psychology, ELTE Eotvos Lorand University, Budapest, Hungary

**Keywords:** internet addiction, online addiction, problematic internet use, Problematic Internet Use Questionnaire, screening instrument, psychometric properties, cross-cultural studies

## Abstract

**Objectives:** To date, there is no reliable instrument which could be used to assess problematic Internet use (PIU) in Lithuania. The nine-item Problematic Internet Use Questionnaire (PIUQ-9) previously validated in multiple countries, could be a potential tool for measuring PIU severity. The main objective of the present study was to explore the psychometric properties of the Lithuanian version of the questionnaire.

**Methods:** A total of 272 students (17% men, mean age 27 ± 9 years) completed the PIUQ-9, the Patient Health Questionnaire (PHQ) and answered questions about the impairment of daily functioning caused by PIU in an online survey.

**Results:** A confirmatory factor analysis indicated that a bi-factor model with one general factor “general problem” and two-specific factors “obsession” and “neglect + control disorder” fitted the data well. The presence of a strong global factor was supported by the common variance index in the bi-factor model indicating that the “general problem” factor explained 67.7% of common variance. The multiple indicators multiple causes (MIMIC) model showed that psychiatric symptoms (β = 0.25) had a moderate, while impairment due to PIU (β = 0.41) had a moderate-to-strong direct effect on the factor “general problem” supporting the construct validity of the scale.

**Conclusion:** The Lithuanian version of the PIUQ-9 has appropriate psychometric properties to be used in measuring PIU severity in student samples.

## Introduction

There is a growing global concern about the public health problems and societal costs of problematic Internet use (PIU), which has an estimated prevalence of 1–27% within the general population (1). PIU is increasingly recognized as disproportionately impacting young people and represents an emerging mental health research challenge (2). Excessive social media use, online streaming, gaming, gambling, online shopping, email checking and pornography viewing are all examples of behaviors that have the potential to cause significant impairment of everyday functioning. Various factors may contribute to PIU including age, gender, socioeconomic status as well as symptoms of mental health conditions or personality traits leading to even more advanced engagement in problematic online behaviors. Furthermore, there is a huge risk that any of these behaviors could have the tendency to increase during and after the COVID-19 quarantine period (3) and may require professional intervention and support. However, it is crucial to have adequate adapted measurements for PIU before developing appropriate tailored interventions.

National health authorities are now expressing concern over the potential impact of PIU. However, there remains both a lack of agreement on what constitutes PIU and scientific data on its prevalence, clinical parameters, and socio-economic burden (2). In order to start answering these important scientific questions, we need an operational definition and reliable tools to measure PIU severity.

While the National Department of Statistics in Lithuania reports that in 2019 ~98% of people aged 16–24 and 95% of people aged 25–34 had been using the Internet daily, there are only few studies investigating PIU prevalence. However, most of the existing studies in European countries which include Lithuania have focused on problematic online behaviors in children and teenagers (4, 5). In a cross-sectional study of 1806 13–18 years old schoolchildren in Lithuania, 9.1% of the study subjects showed markedly expressed symptoms of internet addiction as measured with the Young Diagnostic Questionnaire (6). This particular study also found that symptoms of Internet addiction were also more prevalent among male than female teenagers. Nevertheless, no studies have investigated PIU in young adults and only two reliable questionnaires were used to assess symptoms of Internet addiction in teenagers (4, 5). Therefore, it is crucial to investigate this particular study group of young adults and identify a questionnaire which could be suitable for a specific assessment. A recent publication by Laconi et al. (7) investigated the psychometric properties of the Nine-Item Problematic Internet Use Questionnaire (PIUQ-9) across various different language-based samples of European internet users, highlighting that a brief scale consisting of only nine questions could reliably screen for PIU. The scale has not been used in Lithuania before and its psychometric properties, validated in other cultural backgrounds, show promising results.

Our report attempts to validate a Lithuanian language version of the PIUQ-9 scale. To the best of our knowledge, Lithuania does not a have an adapted measure for PIU severity. Given the brevity of the scale, PIUQ-9 would be useful for researchers, clinicians and educators interested in measuring PIU severity.

## Method

### Procedure

Participants completed an online survey, which provided information about the study (i.e., the study objectives and assurances of anonymity and confidentiality). An online survey was made available from 2019 September until 2019 November using Google Forms. A snowballing technique was used to develop convenience sample targeting students. Firsthand networks of study volunteers and student council assistants were employed by researchers to spread the link. The initial link was shared to Lithuanian University of Health Sciences students using social network services and messenger services such as Facebook or Skype. Researchers encouraged participants to send the link to other students, helping to reach an adequate study sample. However, the engagement rate was not monitored. A formal online consent was provided for each participant to tick before starting the survey. No incentives were given upon completion.

The study received approval from the Bioethics committee and conformed to the principles outlined in the Declaration of Helsinki. The survey included sociodemographic information, the PIUQ-9 questionnaire, and several subscales of the Patient Health Questionnaire (PHQ).

### Measures

Besides collecting basic information on age and gender, we also assessed impairment due to PIU by asking study participants a single question with yes/no answer on whether they thought that internet usage impacted their ability to perform daily activities and/or engage in desired social interactions.

The nine-item version of the Problematic Internet Use Questionnaire (PIUQ-9) was used to assess PIU (8) (Refer to the Lithuanian language version in Appendix A). The PIUQ-9 is a short version of the 18-item Problematic Internet Use Questionnaire [PIUQ; (9)]. Originally, both scales were reported to have three subscales: obsession, neglect, and control disorder. Based on the previous study examining the psychometric properties of the PIUQ-9, the short scale has a bi-factor structure including one “general problem” factor and two-specific factors of “obsession” and “neglect + control disorder.” The severity of PIU is assessed through a five point Likert scale, ranging from “never” to “always/almost always.” The sum score of the questionnaire ranges from nine to 45. A Higher score indicates a higher risk of PIU. The internal consistency of the PIUQ-9 was 0.89 in this particular study sample. As in previous studies (7), all survey questions were translated from English to Lithuanian using the procedure of double back-translation. An experienced psychiatrist (VS) conducted the translation and the back translation was performed by a clinical psychologist (JB). The final consensus was reached by discussing significant differences in the back translation as compared to the original English version.

Psychiatric disorder symptoms were assessed using the Patient Health Questionnaire (PHQ) (10). The PHQ consists of several modules assessing symptoms of several psychiatric disorders, including depressive disorder, anxiety disorders, somatoform disorder and alcohol abuse/dependence. The depression module consisted of nine items questioning depression severity over a 2 weeks period with possible scores ranging from zero (“not at all”) to three (“nearly everyday”). The sum score of depression symptomatology ranged from zero to 27. The anxiety module contained seven questions regarding the severity of anxiety symptom levels during a 4 weeks period. Each question was rated on a scale ranging from zero (“not at all”) to two (“more than half the days”) and resulting in a total score ranging from zero to 14. The somatoform module consisted of 13 items regarding physical problems that bothered individuals over a 4 weeks period. Each item ranged from zero (“not bothered”) to two (“bothered a lot”) resulting in a total score ranging from zero to 26. The alcohol abuse/dependence module contained five questions with answers “yes” and “no” asking about the presence of problematic behaviors related to alcohol use occurring more than once in the past 6 months. The total score for the module ranged from zero to five. For the statistical modeling, a general score for the questionnaire summing up the four aforementioned modules was used in order to evaluate the global severity of any psychiatric symptomatology. The internal consistency of PHQ in the current sample was 0.91.

### Statistical Analysis

The SPSS for Windows, version 17.0 (SPSS Inc., Chicago, Illinois) and Mplus 8 (11) were employed for statistical procedures. All statistical procedures were based on the Laconi et al. study validating the psychometric properties of the PIUQ-9 (7). Using Student's *t*-tests we have also compared the PIUQ-9 scores and impairment to engage in daily activities and social life due to excessive internet use between genders. In brief, four alternative factor structures were tested: (i) the three-factor model proposed in the study by Koronczai et al. (8), (ii) a two-factor model where control disorder and neglect factors were merged into one due to the high correlation, (iii) a bi-factor model comprising a dimension of global severity on which each item is loaded, plus three specific factors on which the items belonging to the obsession, neglect and control disorder were loaded and where the correlations between specific factors were fixed at zero; (iii) a bi-factor model representing a global severity dimension plus two specific factors (obsession and neglect + control disorder). The correlations between specific factors were also fixed at zero. The degree of fit of the factor models were measured through confirmatory factor analysis (CFA) with maximum likelihood estimation with robust standard errors (MLR).

The following fit indices were used to evaluate model fit: the ratio of the chi-square to its degrees of freedom (χ^2^/df ratio), the comparative fit index (CFI), the Tucker-Lewis Index (TLI), the goodness of fit index (GFI), the root mean square error of approximation (RMSEA) and its p close value, and the standardized root mean residual (SRMR). The following values were required to indicate adequate fits: χ^2^/df ratio ≤ 5 and preferably ≤ 2; CFI ≥ 0.95; TLI ≥ 0.95; GFI ≥ 0.9, RMSEA < 0.08, SRMR ≤ 0.10 (12).

The explained common variance (ECV) index was used to measure the degree of unidimensionality in the bi-factor model and the percentage of general factor common variance. Omega and omega hierarchical indices were chosen to measure the ability of the PIUQ-9 scores to estimate the combination of factorial constructs, as well as specific target constructs. The index of construct replicability or H index was used for the evaluation of specific factors (≥0.70 was considered as high).

CFA with covariates or multiple indicators multiple causes (MIMIC) confirmatory factor analysis was performed in the Lithuanian language sample to test the construct validity of the best fitting structure (i.e., the bi-factor-model with two specific factors) by exploring correlations between the general and specific factors and four probable predictors (i.e., age, gender, psychiatric symptoms severity, and impairment due to PIU).

## Results

### Descriptive Statistics

This study consisted of 291 students studying at the Lithuanian University of Health Sciences. Nineteen participants (7%) refused to participate in the study, leaving the total study sample of 272 participants (83% women and 17% men, mean age 27 ± 9 years). Thirty-nine participants (14%) indicated that PIU caused significant impairment in their daily living and/or social activities. Neither the PIUQ-9 scores, nor impairment to engage in daily activities and social life due to excessive internet use differed between genders (*p* > 0.05).

### The Model of the PIUQ-9

The X^2^ test was significant in all four factor models. The bi-factor model comprising two specific factors appeared to be the best fitting model according to the fit indices CFI, TLI, RMSEA, and SRMR (Table 1).

**Table 1:** Confirmatory factor analysis of four measurement models of PIUQ-9.

	**χ^2^**	**df**	***p***	**CFI**	**TLI**	**GFI**	**RMSEA [90% CI]**	**RMSEA p close**	**SRMR**	**SSABIC**
**LTHUANIAN VERSION (*n* = 272)**										
3-factor model	92.5	24	<0.001	0.942	0.914	0.936	0.102 [0.081–0.125]	<0.001	0.056	
2-factor model	124.2	26	<0.001	0.917	0.886	0.890	0.118 [0.098–0.139]	<0.001	0.047	
Bifactor model with three specific factors	66.2	21	<0.001	0.962	0.935	0.959	0.089 [0.065–0.114]	0.004	0.034	6169.4
Bifactor model with two specific factors	54.5	20	<0.001	0.971	0.948	0.970	0.080 [0.055–0.105]	0.027	0.031	6163.3

*PIUQ-9, Problematic Internet Use Questionnaire 9-item version; χ^2^, Chi-square; df, degrees of freedom; CFI, comparative fit index; TLI, Tucker-Levis Index; GFI, goodness-of-fit index; RMSEA, root-mean-square error of approximation; 90% CI, 90% confidence interval of the RMSEA; SRMR, standardized root mean residual; SSABIC, Sample size adjusted Bayesian Information Criteria*.

Factor loadings for the bi-factor model with two specific factors are presented in Table 2. All nine items had significant factor loadings on the global PIU factor. The ninth item for the specific factor “obsession” was found to be non-significant; however, this item had strong significant loadings on the main factor. This was the case for items five, seven, and eight in the specific factor “neglect + control,” as these items were not significant on the specific factor, despite having strong significant loadings on the general problem factor.

**Table 2:** Standardized factor loadings of the bifactor model with two specific factors of PIUQ-9.

	**General factor**	**Specific factors**	
		**Obsession**	**Neglect + control disorder**
**LITHUANIAN VERSION (*n* = 272)**			
Item 3	0.832	0.959	
Item 6	0.733	0.100	
Item 9	0.685	0.024^ns^	
Item 5	0.697		0.014^ns^
Item 8	0.493		0.069^ns^
Item 2	0.742		0.154
Item 4	0.736		0.366
Item 7	0.647		0.048^ns^
Item 1	0.474		0.944

*PIUQ-9, Problematic Internet Use Questionnaire 9-item version. All loadings were significant at p <0.005, except when mentioned ns, not significant*.

According to the ECV index of the bi-factor model, the general problem factor in the PIUQ-9 explained 67.7% of common variance, supporting a strong global factor. Table 3 shows ECV indices of specific factors. Evaluation of the precision of a questionnaire to assess the combination of general and specific factors, and a particular target construct was carried out through the calculation of the omega and omega hierarchical coefficients. For the Lithuanian sample, the specific factor “obsession” omega coefficient was 80.7 and 82.7% for the merged specific factor. The omega hierarchical coefficient for the “obsession” specific factor was 20.9% and in the case of the merged specific factor it was 12.8%. With the specific factors explaining ~13–21% of the PIU score these coefficients are considered significant. The H value above >0.70 (i.e., 0.89) indicated the “neglect + control disorder” to have good construct replicability. The obsession factor had a good replicability as well with the H value of 0.92.

**Table 3:** Indicators of dimensionality and reliability of the bifactor model of PIUQ-9.

	**General factor**	**Specific factors**	
	**General problem**	**Obsession**	**Neglect + control disorder**
**LITHUANIAN VERSION**			
ECV	0.677	0.151	0.172
Ω	0.898	0.807	0.827
Ωh	0.834	0.209	0.128
H	0.900	0.920	0.893

*PIUQ-9, Problematic Internet Use Questionnaire 9-item version; ECV, explained common variance, Ω, omega; Ωh, omega hierarchical; H, H index*.

The MIMIC model shown in Table 4 indicates correlations between the general and specific factors and PIU predictors, such as age, gender, psychiatric symptoms, and impairment due to PIU. Generally, psychiatric symptoms had a moderate direct effect on the general problem factor (β = 0.26). Impairment caused due to PIU had a moderate-to-strong direct effect on the general problem factor (β = 0.41). The effect of psychiatric symptoms on the two specific factors, namely “obsession” and “neglect + control disorder” was also moderate (β = 0.21; β = 0.24, respectively). Furthermore, very similar effects of the impairment due to PIU were observed on the two specific factors (β = 0.39; β = 0.38, respectively).

**Table 4:** Multiple indicators multiple causes (MIMIC) model with standardized coefficients (gender and age were both controlled for in the models).

	**General problem**		**Obsession**		**Neglect + Control disorder**	
	**β**	***p***	**β**	***p***	**β**	***p***
**LITHUANIAN VERSION (*n* = 272)**						
Psychiatric symptoms as measured by Patient Health Questionnaire	0.25	<0.001	0.21	<0.001	0.24	<0.001
Impairment to engage in daily activities and social life due to excessive internet use (NO/YES)	0.41	<0.001	0.39	<0.001	0.38	<0.001
Age	−0.19	<0.001	−0.11	0.037	−0.22	<0.001
*Gender*	0.04	0.439	0.01	0.906	0.06	0.237
*R*^2^	0.37	<0.001	0.28	<0.001	0.35	<0.001

*Gender and age were introduced in the models as control variables. MIMIC model with standardized coefficients*.

## Discussion

The main objective of the present study was to explore the psychometric characteristics of the PIUQ-9. Descriptive results available in our study correspond to the Laconi et al. data (7), for example the mean age of the German sample was 27 ± 10, the mean age of the total sample was 26 ± 9, while the mean age in our sample was 27 ± 9 years. The percentage of men involved in the study dramatically varied between countries in the aforementioned international study (7) ranging from 0.4 to 97% with a mean of 38%. Our study involved a relatively low number of male individuals comprising 17% of the sample. While many of the PIU studies are overrepresented by male subjects (2), our study might better reflect characteristics of Internet use in female students. However, PIUQ-9 scores as well as impairment related to the performance of daily and social activities due to excessive Internet use did not differ between genders.

The mean score of 18.4 ± 6.3 of PIUQ-9 in the Lithuanian sample was very close to the mean score of the Polish sample reported in Laconi et al.'s study (7). Lithuania is culturally closest to Poland, thus it is possible that PIU symptomatology severity corresponds. The percentage of impairment caused by PIU (14%) corresponds to the data on the European PIU prevalence in youth from 11 countries (13). However, the impairment percent in our study is slightly higher than in the Lithuanian study investigating schoolchildren (6). Several reasons might explain the discrepancy. First, students are the group with the most expressed Internet consumption. Secondly, problematic use and true clinical impairment due to PIU while overlapping are still two different concepts, which measured with different questionnaires might produce discrepancy. Thus, it is crucial to have a questionnaire which could reliably identify PIU symptomatology in prevalence studies.

Our results have confirmed good fit indices for the bi-factor model, with one “general problem” factor and two specific factors of “obsession” and “neglect + control disorder.” Psychiatric symptoms had a moderate direct effect on the general problem factor in the Lithuanian sample, showing good construct validity of the scale. Impairment related to the performance of daily and social activities due to excessive Internet use was a moderate-to-strong predictor of PIU.

The major limitation of the present study is the cross-sectional design, thereby precluding us from making causal assumptions. The Lithuanian sample consisted of students attending one university and was a comparatively small sample size. Furthermore, the gender distribution of the sample was skewed, with a considerably higher proportion of female students. Thus, the results may not represent general or clinical populations and may be subject to selection bias. Questions on educational level (14), and socioeconomic status were not included into our survey, limiting a broader scope of the PIU context in students. While we changed the time spent online covariate with the perceived impairment one, we consider that not including this criterion is a significant limitation of this study. Differentiating time spent online on weekend might have further contributed to the general factor of the questionnaire (7).

To the best of our knowledge this is the first study validating the PIUQ-9 questionnaire in the Lithuanian language. The Lithuanian version of the PIUQ-9 has demonstrated appropriate psychometric characteristics and is suitable for screening PIU in university students. Self-reported perceived impairment related to Internet usage is a strong predictor of general PIU psychopathology. Our current study adds PIUQ-9 as a validated tool for clinicians and educators to screen for the risk and severity of PIU. During a time with an increased Internet usage due to COVID-19 requirements to self-isolate or study remotely, PIUQ-9 could be a potential tool in measuring PIU severity. Future longitudinal studies should observe PIU changes over time and establish a cut-off threshold to define individuals who are at possible risk of PIU and therefore should be advised to seek treatment.

In summary, the PIUQ-9 has good psychometric characteristics and can be applied in research, as well as for educational and clinical purposes, to screen for the risk and severity of PIU.

## Data Availability Statement

The raw data supporting the conclusions of this article will be made available by the authors, without undue reservation.

## Ethics Statement

The studies involving human participants were reviewed and approved by Lithuanian University of Health Sciences Bioethics Center. The patients/participants provided their written informed consent to participate in this study.

## Author Contributions

JB, VS, ZD, and OK conceived and designed the study. VS and JB were responsible for data collection and evaluation. Statistical analyses were performed by AP. JB prepared the manuscript. All authors contributed to the article and approved the submitted version.

## Conflict of Interest

In the past several years, JB has been serving as a consultant for Cogstate, Ltd. The remaining authors declare that the research was conducted in the absence of any commercial or financial relationships that could be construed as a potential conflict of interest.
