# Supplementary material for: Psychometric Properties of the Nine-Item Problematic Internet Use Questionnaire (PIUQ-9) in a Lithuanian Sample of Students
Source: Front Psychiatry. 2020 Nov 12;11:565769. doi: 10.3389/fpsyt.2020.565769 (PMC7688508; doi:10.3389/fpsyt.2020.565769)
Supplement: Supplementary file 1 [file Data_Sheet_1.docx]

Supplementary Material

**APPENDIX A. Lithuanian version of PIUQ-9**

**Probleminio interneto naudojimo klausimyno devynių klausimų versija (PIUQ-9)**

Toliau Jūs perskaitysite teiginius apie naudojimąsi internetu. Naudodamiesi skale nuo 1 iki 5, nurodykite, kiek kiekvienas teiginys Jums tinka:
1 = niekada
2 = retai
3 = kartais
4 = dažnai
5 = visada/beveik visada

**Kaip dažnai...**

1. Jūs jaučiatės, kad turėtumėte sumažinti leidžiamo laiko internete kiekį?
2. Jūs apleidžiate namų ruošos darbus, kad galėtumėte praleisti daugiau laiko internete?
3. Jūs jaučiatės įsitempęs, susierzinęs ar patiriate stresą jei negalite naudotis internetu tiek, kiek jūs norite?
4. Būna taip, kad Jūs norėtumėte sumažinti leidžiamo laiko internete kiekį, tačiau jums nepavyksta?
5. Jūs leidžiate laiką internete vietoj to, kad miegotumėte?
6. Jūs jaučiatės įsitempęs, susierzinęs ar patiriate stresą jei negalite naudotis internetu keletą dienų?
7. Jūs bandote nuslėpti, kiek laiko praleidžiate internete?
8. Žmonės jūsų gyvenime skundžiasi, kad Jūs per daug laiko praleidžiate internete?
9. Būna taip, kad jūs jaučiate depresiją, būnate blogos nuotaikos ar būnate nervingas kai nesate internete, o šie jausmai dingsta tuomet, kai Jūs vėl grįžtate prie interneto?
